# Antioxidant Effects of Salidroside in the Cardiovascular System

**DOI:** 10.1155/2020/9568647

**Published:** 2020-09-26

**Authors:** Siyu Sun, Qinhui Tuo, Dongxu Li, Xiulong Wang, Xuefang Li, Yiyue Zhang, Guoan Zhao, Fei Lin

**Affiliations:** ^1^The Cardiovascular Research Center, The First Affiliated Hospital of Xinxiang Medical University, Xinxiang, Henan 453003, China; ^2^The School of Pharmacy, Hunan University of Chinese Medicine, Changsha, Hunan 410208, China

## Abstract

Cardiovascular disease is one of the main human health risks, and the incidence is increasing. Salidroside is an important bioactive component of *Rhodiola rosea* L., which is used to treat Alzheimer's disease, tumor, depression, and other diseases. Recent studies have shown that salidroside has therapeutic effects, to some degree, in cardiovascular diseases via an antioxidative mechanism. However, evidence-based clinical data supporting the effectiveness of salidroside in the treatment of cardiovascular diseases are limited. In this review, we discuss the effects of salidroside on cardiovascular risk factors and cardiovascular diseases and highlight potential antioxidant therapeutic strategies.

## 1. Introduction

Cardiovascular diseases (CVDs) are the most common cause of mortality globally among all diseases. Heart attack and stroke are the leading causes of CVDs-related deaths. Individuals with CVDs or with risk factors for CVDs, such as diabetes and hyperlipidemia, need early detection and appropriate medical interventions [1]. Owing to the complex mechanisms underlying CVDs, in addition to conventional medicines, the identification of natural herbal products with multiple targets is an important research goal. Salidroside (SAD) is a polyphenolic compound isolated from *Rhodiola rosea* L. According to Modern Practical Materia Medica, *R. rosea* L. has the following effects: (1) central inhibitory effect, (2) antifatigue effect, (3) promotion of cardiovascular function, (4) anti-inflammatory effect, (5) hypoglycemic effect, (6) antiperoxidation effect, and (7) antiradiation effect. SAD, an important bioactive component of *R. rosea* L., is effective for the treatment of Alzheimer's disease, depressive disorder, tumor, and CVDs [2–5]. In particular, accumulating studies show that SAD plays a protective role by suppressing cardiovascular risk factors and the development of coronary heart disease, heart failure, stroke, and pulmonary hypertension. This review provides a systematic overview of the effects and mechanisms of action of SAD in CVDs. In particular, we summarize evidence for the antioxidant effects of SAD, its relationships with risk factors for CVDs (e.g., obesity and aging), and its effects in various CVDs (e.g., atherosclerosis and stroke).

## 2. Antioxidant Effects of SAD

Oxidative stress refers to the imbalance between oxidation and antioxidation *in vivo*, and it is associated with aging and various diseases. Reactive oxygen species (ROS) overproduction is the main reason for this imbalance. Mitochondria are a major source of intracellular ROS. The antioxidant system can effectively remove the ROS produced during metabolism and protect biological macromolecules against oxidative damage by ROS, including superoxide dismutase (SOD), glutathione peroxidase (GSH-Px), and catalase (CAT) [6, 7]. Accordingly, mitochondrial damage or a decrease in antioxidant enzyme synthesis results in excessive ROS production. *R. rosea* L. ethanol extract, which has high levels of phenolic compounds, particularly SAD, has strong antioxidant activity, as determined by DPPH, ABTS, and FRAP assays [8]. Further studies have shown that SAD not only inhibits ROS production by the regulation of mitochondrial biogenesis but also inhibits ROS-mediated CVDs by increasing the activity of the antioxidant enzymes SOD and GSH-Px [9, 10]. Finally, SAD has antioxidant effects *in vivo* and *in vitro*.

## 3. Cardiovascular Risk Factors

CVDs are the result of long-term interactions among many adverse factors. Risk factors include hyperlipidemia, smoking, drink, diabetes, obesity, aging, and genetic factors. Active prevention and treatment of these risk factors are essential to reduce the incidence of CVDs. Extensive research has shown that SAD influences cardiovascular risk factors, as summarized below.

### 3.1. Obesity and Diabetes

Epidemiological analyses have proven a clear relationship among diabetes, obesity, and CVDs. Notably, the progression from obesity to diabetes associated with an abnormal lipid profile is strongly correlated with insulin resistance. As highlighted in the literature, the relationships among obesity, abnormal lipid profiles, insulin resistance, and diabetes contribute to the occurrence and development of CVDs [11, 12]. Various studies have evaluated the use of SAD for the treatment of obesity, insulin resistance, and diabetes. Wang et al. find that 50 mg/kg/day SAD for 48 days can significantly repress the elevation of body weight and adipogenesis in epididymal white adipose tissues by reducing food intake. It also attenuates the levels of triglycerides and total cholesterol in the liver. SAD can decrease the levels of total triacylglycerides, total cholesterol, low-density lipoprotein cholesterol, and high-density lipoprotein cholesterol in the plasma [13, 14]. Insulin resistance is involved in the pathogenesis of these disorders and may benefit from intervention with SAD. As expected, SAD reduces blood glucose and serum insulin levels and increases sensitivity to insulin. These effects may be mediated by AMPK/SIRT1 signaling and the mitochondria-related AMPK/PI3K/Akt/GSK3*β* pathway [15, 16]. Insulin resistance is responsible for the development of type 2 diabetes mellitus. Recent studies have shown that SAD stimulates glucose uptake, regulates hepatic gluconeogenesis and lipid metabolism, and improves *β*-cell survival in the treatment of diabetes [17, 18].

SAD has beneficial effects on vascular diastolic dysfunction via the soluble guanylyl cyclase pathway in the Goto–Kakizaki model of type 2 diabetes [19]. SAD (1–10 *μ*g/ml) has a protective effect on high glucose-induced endothelial cell injury by activating the Ca/CaM/CAMKII/eNOS pathway [20]. SAD prevents advanced glycation end-product-induced endothelial dysfunction, and its effects may be attributed, in part, to the induction of HO-1 and the attenuation of phosphorylated NF-kB p65 [21]. In addition, SAD ameliorates diabetic nephropathy and antihyperalgesic activity and prevents cognitive impairment in a rat model of diabetes [22–24]. Accordingly, we expect SAD to be useful for the treatment of lifestyle-related diseases, such as hyperlipidemia, exogenous obesity, and diabetes.

### 3.2. Hyperlipidemia

Hyperlipidemia is a risk factor for CVDs. A large number of studies have proven that the interplay between lipids and immune cell infiltration is the main cause of the formation of vascular atherosclerosis (AS). AS begins with the accumulation and oxidation of subendothelial cholesterol in blood vessels. This subsequently stimulates the production of innate and acquired immunity and causes chronic inflammation of the vascular wall [25]. Since the theory of oxidative modification of LDL in AS was initially proposed 30 years ago, oxidized low-density lipoprotein (ox-LDL) has been used as a stimulator of AS. The protective effects of SAD have been demonstrated in ox-LDL-injured HUVECs, and these effects may be related to the inhibition of oxidative stress inhibition and restoration of mitochondrial dysfunction by activating the AMPK/SIRT1 pathway [26]. Further research has shown that SAD also protects against ox-LDL-induced endothelial injury by promoting autophagy via the SIRT1-FoxO1 signaling pathway [27].

### 3.3. Nonalcoholic Fatty Liver Disease

Increasing studies indicate that nonalcoholic fatty liver disease (NAFLD) is independently associated with the development of carotid intima-media thickening and plaques and coronary artery calcification, with a 65% increase in CVDs events [28]. Consistent with the effects of SAD on obesity, lipid metabolism, insulin resistance, and diabetes, it has a regulatory effect on high-fat diet-induced NAFLD. In particular, SAD has an anti-NAFLD effect via the inhibition TRPM2 ion channel activation, regulation of AMPK-dependent TXNIP/NLRP3 pathways, insulin signaling pathway, and gut microbiota-bile acid-farnesoid X receptor axis [29–32].

### 3.4. Aging

It is well-known that aging is one of the key factors in the occurrence of CVDs. Antioxidants have beneficial effects in aging-associated CVDs [33]. Recently, the Chinese medicinal herb *Rhodiola* has been reported to have antiaging activity in Alzheimer's disease, Parkinson's disease, CVDs, and so on [5]. SAD, as its main component with antioxidant effects, prolongs the lifespan and delays the onset of age-related biomarkers [34]. In addition, SAD attenuates endothelial cellular senescence and inhibits the vascular aging response [35].

## 4. Cardiovascular Diseases

### 4.1. Coronary Heart Disease

#### 4.1.1. Atherosclerosis

AS is the main pathological basis of coronary heart disease, cerebral atherosclerotic infarction, and peripheral vascular disease. The pathogenesis of AS involves several key factors: (1) endothelial cell injury, (2) lipid accumulation at the injury site, (3) internalization of lipids by monocytes and foam cell formation, and (4) inflammatory factor-induced proliferation of vascular smooth muscle cells. The protective effects of SAD have been investigated in female LDL receptor knockout (LDLr^−/−^) mice treated with a high-fat diet to induce AS. SAD treatment (50 mg/kg/day for 8 weeks) clearly reduced the plaque area of the aortic arch by lowering lipids and anti-inflammatory effects [36]. Furthermore, NLRP3-related pyroptosis might be another mechanism by which SAD decreases AS plaque formation [37]. The antiatherosclerotic effects of SAD protect endothelial function by promoting nitric oxide (NO) production, which is associated with mitochondria depolarization and the subsequent activation of the AMPK/PI3K/Akt/eNOS pathway [38].

Endothelial dysfunction plays important roles in CVDs, including AS. Oxidative stress, the renin-angiotensin system, ox-LDL, and homocysteine are the main causes of endothelial injuries [39]. Consequently, endothelial cells could be targets of SAD to protect against AS. Pretreatment of cells with SAD significantly reduces endothelial injury in a process mediated by the regulation of oxidation stress signaling pathways, such as the AMPK pathway, mTOR pathway, and Nrf2 pathway, in response to stimulation by hydrogen peroxide (H_2_O_2_) [40–42]. Using another model of homocysteine stimulation, SAD improved NO bioavailability, stimulated mitochondrial biogenesis, inhibited ROS production, and regulated the ER-stress pathway to protect endothelial cell function [9, 43, 44]. In addition, SAD exerts angiogenic and cytoprotective effects via the Akt/mTOR/p70S6K and MAPK signaling pathways in human bone marrow-derived endothelial progenitor cells [45].

#### 4.1.2. Myocardial Ischemia

Myocardial infraction (MI) is a CVDs caused by persistent ischemia and hypoxia of the coronary artery. Ischemia/reperfusion (I/R) injury is an important complication during the treatment of MI in clinical settings. However, effective cardioprotective therapies are lacking for MI and I/R injury. Some scholars have recently proposed that focusing on the rational combination of judiciously selected and multitargeted therapies may be effective [46]. Multitarget mechanisms underlying the effects of SAD under MI have been identified. Recent studies have shown that SAD is a multitarget drug. On the one hand, SAD protects against LPS-induced myocardial injury *in vivo* and hydrogen peroxide-induced myocardial cell injury i*n vitro* via the activation of the PI3K/Akt pathway [47, 48]. On the other hand, SAD protects against hypoxia-induced myocardial cell death and promotes cardiac angiogenesis in acute MI rats by upregulating HIF-1*α* and the VEGF-mediated pathway [49, 50]. In addition, SAD inhibits apoptosis by restoring the tricarboxylic acid cycle and by the preservation of mitochondrial integrity [51, 52]. Finally, SAD attenuates isoproterenol-induced acute MI by the regulation of the Nox/NF-*κ*B/AP1 pathway [53]. These studies support the role of SAD as a potential treatment for ischemic heart disease.

I/R injury refers to ischemia and subsequent reperfusion injury in acute MI. Recently, the effects and mechanisms of action of traditional Chinese medicine on I/R injury have been gradually characterized [54]. SAD is effective in preventing I/R injury. By pretreating rats with 50 mg/kg SAD, Xu et al. found that SAD activates the PI3K/Akt pathway and reduces apoptosis in cardiomyocytes, which in turn inhibits I/R injury [55]. In cell experiments, myocardial cells were induced by hypoxia/reoxygenation to mimic I/R injury, and the injury effect was inhibited by SAD pretreatment, as evidenced by the suppression of apoptosis, an increase in *N*-acetylglucosamine linkage to cellular proteins, and the activation of Akt signaling [56–58].

### 4.2. Stroke

Similar to MI, increasing evidence shows that SAD and its analogues reduce ischemic stroke and cerebral I/R injury in adult rats. In general, the mechanisms underlying the neuroprotective effects of SAD may involve three pathways. First, SAD inhibits the inflammatory responses in multiple ischemic stroke processes, as indicated by the reduction of LPS-induced BV2 microglial cell mobility via NF-B and MAPK signaling, the reduction of inflammatory effects via PI3K/Akt signaling after permanent middle cerebral artery occlusion or cerebral I/R injury, and the induction of primary microglia from the M1 phenotype to M2 phenotype [59–62]. Second, protective effects of SAD against ischemic stroke are related to endothelial function. The intraperitoneal administration of SAD before middle cerebral artery occlusion improves human brain microvascular endothelial cell activity by activating the PI3K/Akt pathway. Furthermore, SAD alleviates brain ischemic injury, and I/R injury caused blood-brain barrier injury by delayed tPA treatment and the inhibition of tumor necrosis factor-alpha [63, 64]. Third, SAD regulates mitochondrial function against exertional heat stroke-induced organ damage in a rat model [65]. Recently, increasing SAD analogues have been found to have the same neuroprotective effects. For example, 2-(4-methoxyphenyl)ethyl-2-acetamido-2-deoxy-*β*-D-pyranoside (GlcNAc-Sal) confers neuroprotective effects via the regulation of local glucose metabolism by increasing glucose uptake, *O*-GlcNAcylation, and elevating GLUT3 expression in a rat model of cerebral ischemic injury [66–68]. Further research has shown that GlcNAc-Sal prevents brain I/R injury and suppresses mouse hippocampal HT22 cell apoptosis by reducing ROS generation, NO production, and the expression of caspase-3 [69]. In addition, the SAD metabolite *p*-tyrosol at a dose of 20 mg/kg also attenuates neuronal damage in the hippocampus as well as lipid peroxidation in brain tissue in animals subjected to global cerebral ischemia with reperfusion [70]. Taken together, SAD could prevent stroke and exert neuroprotective effects via multiple mechanisms.

### 4.3. Cardiac Function Injury in Exhaustive Exercise

Exercise training can maintain health, but exhausting exercise leads to a reduction in cardiac function. There is experimental evidence that SAD has a protective effect against exercise-induced decreases in cardiac function. The protective effects of SAD have been investigated in rats trained with exhaustive swimming. SAD treatment evidently decreases the levels of brain natriuretic peptide, cardiac troponin I, and ROS in the serum [71]. It also decreases the level of CK-MB, which is mainly used to diagnose acute myocardial injury [72]. Further research has shown that the underlying mechanisms include antioxidative stress by MAPK signal transduction and improved mitochondrial respiratory function by the Nrf2 signaling pathway [71, 72].

### 4.4. Pulmonary Hypertension

Pulmonary hypertension refers to a hemodynamic and pathophysiological state in which pulmonary arterial pressure increases beyond a certain threshold, leading to right heart failure. Pulmonary hypertension is a common and frequently occurring disease with high rates of morbidity and mortality. Increasing evidence shows that *Rhodiola* may be used for the treatment of pulmonary hypertension [73]. The abnormal proliferation and apoptotic resistance of pulmonary vascular smooth muscle cells are major causes of pulmonary hypertension. *Rhodiola* and SAD have antipulmonary hypertension effects, mainly by inhibiting cell proliferation and promoting cell apoptosis. The main mechanisms include the regulation of the adenosine A2a receptor-related mitochondria-dependent apoptosis pathway, AMPK pathway, and AKT pathway [74–76]. In addition, SAD can also reduce blood pressure in diabetic rats and has a dual protective role in diabetes and hypertension [77].

### 4.5. Drug-Induced Cardiotoxicity and Myocarditis

Anthraquinones are widely used for the treatment of acute and chronic leukemia, malignant lymphoma, breast cancer, and other solid tumors. However, their clinical applications are limited by cardiotoxicity. SAD inhibits this cardiotoxicity. Wang et al. found that SAD effectively protects cardiomyocytes against doxorubicin-induced cardiotoxicity by suppressing excessive oxidative stress and activating a Bcl2-mediated survival signaling pathway [78]. Zhang et al. found that SAD has a protective effect against epirubicin-induced early left ventricular regional systolic dysfunction in patients with breast cancer [79].

Myocarditis is often caused by the immune response after viral infection. Wang et al. found that SAD possesses antiviral activity *in vivo* and *in vitro* by an antioxidant effect and the inhibition of cytokine expression [80]. Similarly, SAD reduces LPS-induced myocardial depression in sepsis by regulating the inflammatory response [81]. Therefore, it is a candidate therapeutic agent for myocarditis.

## 5. Molecular Targets of SAD in CVDs

**Figure 1:**
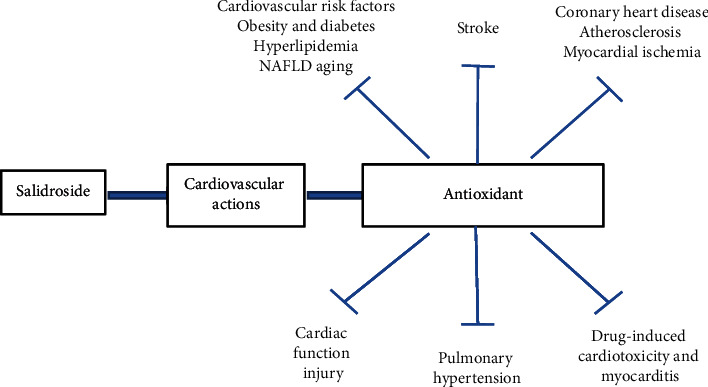
Cardiovascular actions of salidroside.

**Figure 2:**
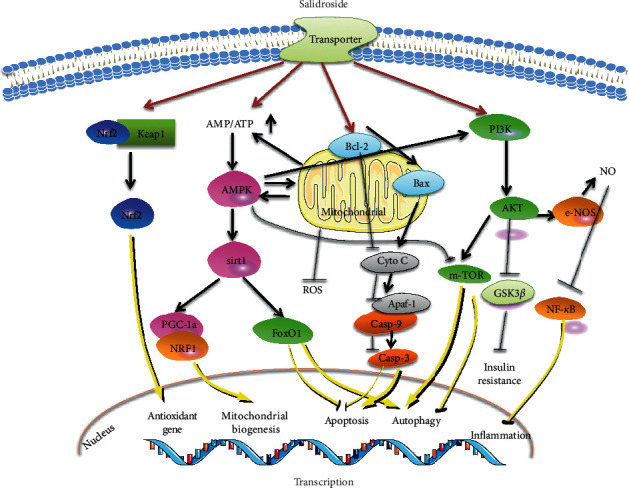
Molecular targets of salidroside.

In light of emerging evidence for the cardiovascular benefits of SAD (Figure 1), we discuss the major molecular targets in detail (Figure 2). (1) AMPK signaling pathway. AMPK plays a major role in regulating the cellular energy balance. The occurrence and development of CVDs are closely related to the disruption of cellular energy metabolism. Therefore, AMPK is an important molecular determinant of CVDs [82]. In a review of recent literature, Zheng concluded that AMPK is an important target of SAD in diabetes [17]. Furthermore, AMPK can regulate autophagy, mitochondrial function, apoptosis, and inflammatory responses by interacting with PI3K/Akt, mTOR, and SIRT1 in endothelial cells [26, 38, 40]. (2) PI3K/Akt signaling pathway. Akt can widely regulate cell proliferation, survival, growth, migration, and other physiological functions. SAD activation of the PI3K/Akt signaling pathway and downstream targets, such as mTOR and GSK3*β*, determines its function in cardiovascular processes [15, 45]. (3) Mitochondria-dependent signaling pathway. Traditional Chinese medicine used to treat CVDs is closely related to the regulation of mitochondrial function [83]. SAD results in specific mitochondrial depolarization and therefore is able to potently regulate the mitochondria-dependent signaling pathway, modulate mitochondrial-mediated apoptosis, and decrease ROS production [38, 52]. (4) Nrf2 signaling pathway. Nrf2 is an important transcription factor in the regulation of oxidative stress and a central regulator in the maintenance of intracellular redox homeostasis [84]. SAD, as an antioxidant, can induce Nrf2 nuclear translocation, activate the expression of Nrf2-regulated antioxidant enzyme genes, and decrease the levels of intercellular ROS [42].

## 6. Discussion

Based on the research summarized in this review, we conclude that SAD has an anti-CVDs effect via an antioxidative mechanism. However, the clinical use of SAD requires additional experimental studies. For example, the bioavailability of SAD is not clear. Guo et al. have reported that SAD is rapidly metabolized to *p*-tyrosol after i.v. administration (50 mg/kg) in rats and has the highest concentration in the heart. However, 64.00% of the total dose is excreted through the urine in the form of SAD [85]. Owing to the lack of detailed bioavailability, pharmacology, and toxicology experiments, an appropriate clinically recommended dose has not been established, limiting its clinical applications. Additionally, SAD may have effects on cardiovascular drug metabolism. The main enzymes for metabolizing drugs in humans are cytochrome P450 proteins (CYP). *R. rosea L.* inhibits CYP2C9 [86]. Losartan, glimepiride, and other drugs commonly used to treat hypertension and diabetes are mainly metabolized by CYP2C9 enzymes. The inhibition of these metabolic enzymes may increase the long-term efficacy of drugs. However, it may also increase hepatotoxicity. Finally, there are few clinical trials of SAD in CVDs. Although Dazhu Hongjingtian injection (YBZ11852006) promotes blood circulation and reduces blood stasis, it is mainly used for the treatment of stable exertion angina pectoris in coronary heart disease. Traditional Chinese medicine syndrome differentiation for heart blood stasis is based on the following symptoms: chest pain, colic, immovable, pain-induced shoulder back and medial arm, chest tightness, palpitation restlessness, dark lips and tongue, and fine pulse. However, to confirm that SAD is the main component underlying its functions, more experimental research is needed.

## 7. Conclusion

CVDs are the result of the long-term accumulation of multiple factors. Therefore, it is necessary to develop multitargeted treatments. Overall, salidroside has an established chemical structure and metabolites with an efficient heart-targeting effect. It also affects cardiovascular diseases via anti-inflammatory, antioxidant, and antiapoptotic effects, including atherosclerotic coronary heart disease, MI, and stroke. Therefore, SAD is a potential anticardiovascular drug. However, in view of the lack of clarity regarding its mechanism of action, additional experimental research is needed for clinical applications.
